# Vacuoles, E1 enzyme, X-linked, autoinflammatory, and somatic syndrome in the intensive care unit: a case report

**DOI:** 10.1186/s13256-023-04034-5

**Published:** 2023-07-22

**Authors:** Félicie Belicard, Nicolas Belhomme, Simon Bouzy, Clémence Saillard, Fabienne Nedelec, Jean-Baptiste Mear, Samuel Ardois, Cedric Pastoret, Florian Reizine, Christophe Camus, Benoit Painvin

**Affiliations:** 1 Service de Réanimation Médicale et des Maladies Infectieuses, Centre Hospitalier Universitaire de Rennes, Hôpital Pontchaillou, 2 rue Henri le Guilloux, 35033 Rennes Cedex 9, France; 2 Internal Medicine Department, Centre Hospitalier Universitaire de Rennes, Hôpital Pontchaillou, 2 rue Henri le Guilloux, 35033 Rennes Cedex 9, France; 3 Hematology Laboratory Department, Centre Hospitalier Universitaire de Rennes, Hôpital Pontchaillou, 2 rue Henri le Guilloux, 35033 Rennes Cedex 9, France; 4 Dermatology Department, Centre Hospitalier Universitaire de Rennes, Hôpital Pontchaillou, 2 rue Henri le Guilloux, 35033 Rennes Cedex 9, France; 5 Hemostasis Laboratory Department, Centre Hospitalier Universitaire de Rennes, Hôpital Pontchaillou, 2 rue Henri le Guilloux, 35033 Rennes Cedex 9, France; 6 Hematology Department, Centre Hospitalier Universitaire de Rennes, Hôpital Pontchaillou, 2 rue Henri le Guilloux, 35033 Rennes Cedex 9, France

**Keywords:** VEXAS syndrome, Vacuoles, Autoinflammatory disease, Cardiac arrest, ICU, Sweet’s syndrome

## Abstract

**Background:**

Vacuoles, E1 enzyme, X-linked, autoinflammatory, and somatic syndrome is a newly discovered inflammatory disease affecting male subjects, for which few data exist in the literature. Here, we describe the case of a patient with known Sweet’s syndrome admitted to the intensive care unit and for whom a vacuoles, E1 enzyme, X-linked, autoinflammatory, and somatic syndrome was diagnosed, allowing for appropriate treatment and the patient’s discharge and recovery.

**Case presentation:**

A 70-year-old male White patient was hospitalized in the intensive care unit following an intrahospital cardiac arrest. History started a year before with repeated deep vein thrombosis and episodes of skin eruption compatible with Sweet’s syndrome. After a course of oral steroids, fever and inflammatory syndrome relapsed with onset of polychondritis, episcleritis along with neurological symptoms and pulmonary infiltrates. Intrahospital hypoxic cardiac arrest happened during patient’s new investigations, and he was admitted in a critical state. During the intensive care unit stay, he presented with livedoid skin lesions on both feet. Vasculitis was not proven; however, cryoglobulinemia screening came back positive. Onset of pancytopenia was explored with a myelogram aspirate. It showed signs of dysmyelopoiesis and vacuoles in erythroid and myeloid precursors. Of note, new deep vein thrombosis developed, despite being treated with heparin leading to the diagnosis of heparin-induced thrombocytopenia. The course of symptoms were overlapping multiple entities, and so a multidisciplinary team discussion was implemented. Screening for* UBA1*-mutation in the blood came back positive, confirming the vacuoles, E1 enzyme, X-linked, autoinflammatory, and somatic syndrome. Corticosteroids and anti-IL1 infusion were started with satisfactory results supporting patient’s discharge from intensive care unit to the internal medicine ward.

**Conclusions:**

Vacuoles, E1 enzyme, X-linked, autoinflammatory, and somatic syndrome should be suspected in male patients presenting with inflammatory symptoms, such as fever, skin eruption, chondritis, venous thromboembolism, and vacuoles in bone marrow precursors. Patients with undiagnosed vacuoles, E1 enzyme, X-linked, autoinflammatory, and somatic syndrome may present with organ failure requiring hospitalization in intensive care unit, where screening for *UBA1* mutation should be performed when medical history is evocative. Multidisciplinary team involvement is highly recommended for patient management, notably to start appropriate immunosuppressive treatments.

## Background

Vacuoles, E1 enzyme, X-linked, autoinflammatory, and somatic (VEXAS) syndrome is a newly discovered medical entity that belongs to the field of inflammatory diseases and affects only male patients [1]. It is linked to a genetic mutation named* UBA1*. Symptoms range from fever, fatigue, chondritis, cytopenia, and skin lesions [1, 2]. Vacuoles in myeloid and erythroid precursors in a bone marrow aspirate are specific to the VEXAS syndrome. Treatment is based on corticosteroids and immunosuppressive drugs [3–5]. Medical management and appropriate treatment need multidisciplinary team involvement. Our case describes a patient with complex inflammatory medical history who presents with an intrahospital cardiac arrest and for whom the diagnosis of VEXAS syndrome is established during the intensive care unit (ICU) course.

## Case presentation

In October 2019, a 70-year-old male White patient, with a medical history of type 2 diabetes and prostate cancer in remission, no psychosocial background, and no family medical history, developed a right leg deep vein thrombosis (DVT). He initially received the oral anticoagulant, rivaroxaban, for three months. A month later, persistent fever at 38.5 °C was noted. Furthemore, a skin rash on the four limbs, predominant on the back of the hands and made of sharp-edged infiltrated papuples that were initially non-itchy and painless, were noticed by the patient while at home.

**Fig. 1:**
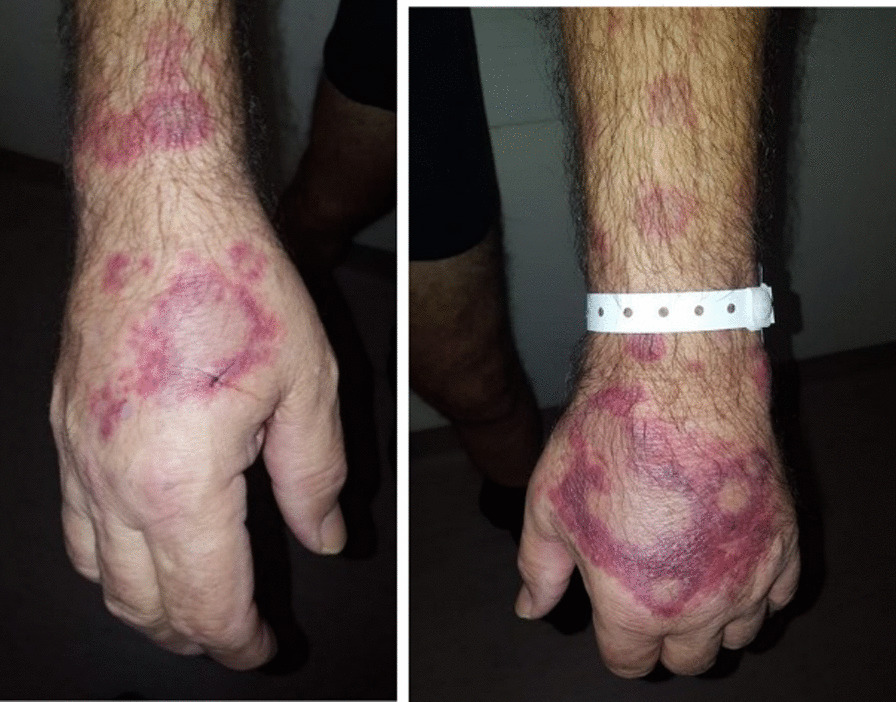
First skin lesions of the patient, with skin biopsy confirming Sweet’s syndrome

By the end of the year, a rapid centrifugal extension of the skin lesions appeared on the four limbs, with erythematous edges and a purplish centers, leading to a quick referral to the dermatology unit of the University Hospital of Rennes, France. A diagnosis of Sweet’s syndrome, also known as acute febrile neutrophilic dermatosis, was confirmed by skin biopsy [6] (Fig. 1). Because of the association of Sweet’s syndrome and the recent deep vein thrombosis, cancer was ruled out: a chest and abdomen computed tomography (CT) scan was interpreted as neoplasia-free. Other investigations showed an inflammatory syndrome [C-reactive protein (CRP) was 187 mg/L] and polyclonal hypergammaglobulinemia at 20.9 g/L. Screening for JAK-2 mutation was negative. Screening for viral and bacterial infection was also negative. Treatment for Sweet’s syndrome consisted of an oral corticosteroids course as follows: prednisone 50 mg/day for 7 days and then 25 mg/day for 7 days, followed by 10 mg/day for 10 days and then treatment was stopped.

The patient was discharged 3 days after corticosteroid initiation, with rapid improvement of his skin condition. However, steroids tapering resulted in multiple skin eruption recurrences in January and April 2020, hindering complete drug withdrawal until November 2020.

By the end of 2020, asthenia, weight loss, and recurrence of skin eruption, along with palpebral edema, led to a new hospitalization in the Dermatology Department of the University Hospital of Rennes.

Admission tests revealed an inflammatory biological syndrome with a CRP at 257 mg/L. During the hospitalization, polychondritis was suspected on the basis of laryngeal pain, helix pain of the left ear, and sudden unilateral hearing loss that developed in less than a month. Right eye episcleritis along with chemosis and left eyelid edema were diagnosed concomitantly. Swallowing disorders and dysphagia developed progressively. An esophagogastroduodenoscopy was normal and a nasofibroscopy found an aspecific retro-cricoarytenoid edema.

During this hospitalization, the patient presented with new neurological symptoms as followed: progressive sensitive disorders on both feet with hyperesthesia, and proprioceptive anomaly. Cerebral and cervical spinal cord magnetic resonance (MRI) were normal and lumbar punction found no abnormalities: absence of cells, proteinorachia of 0.58 g/L, normoglycorachia, no microorganisms, no malignant cells, and negative serologic testing for Lyme disease. Autoimmune encephalitis antibodies testing (antineurones and anti-NMDA receptor) in the cerebral spinal fluid was negative.

Other symptoms included muscle pain associated with amyotrophia. To document these features, a positron emission tomography (PET) scan showed diffuse and heterogeneous muscular metabolic hypersignal and a MRI confirmed a diffuse muscular short tau inversion recovery (STIR) hypersignal. Autoimmune myositis was suspected. The diagnosis was ruled out on the basis of non-elevated creatine phosphokinase (CPK) enzymes and negative antiextractable nuclear antigen (anti-ENA) autoantibodies, even though muscular biopsy could not have been performed due to the following evolution. Myasthenia gravis specific autoantibodies (anti-MuSK and antiacetylcholine receptor) were also negative.

Soon after, new deep vein thrombosis (DVT) appeared on the right humeral vein with extension to the right axillary and subclavian veins, despite appropriate use of rivaroxaban since the finding of DVTs in June 2020.

Tests for thrombophilia found a mild antithrombin III deficiency (66%, with a normal value being over 80%) and positivity of one antiphospholipid antibody out of three, with negative controls 3 weeks later. Switch from rivaroxaban to subcutaneous tinzaparin sodium was followed by the onset of new DVT of the right leg. Hence, tinzaparin was stopped, and intravenous heparin was started.

A chest CT scan was planned on 10 January 2021 because of recent worsening respiratory function. While lying on the CT scan table, the patient developed non-shockable cardiac arrest. The medical radiology team deployed an automatic external defibrillator, which did not deliver any shock.

The rescue team provided emergency measures, which consisted of cardiopulmonary resuscitation (CPR) for 12 minutes and intravenous injection of 4 mg of epinephrine. Rescue team proceeded to orotracheal intubation and the patient was transferred to our medical ICU. Initial 24 hour sedation with controlled body temperature at 36 °C were conducted according to international guidelines [7]. Awakening occurred during weaning from sedation. The patient was able to answer orders and had no neurological deficit. Yet, his confusion was persistent.

**Fig. 2:**
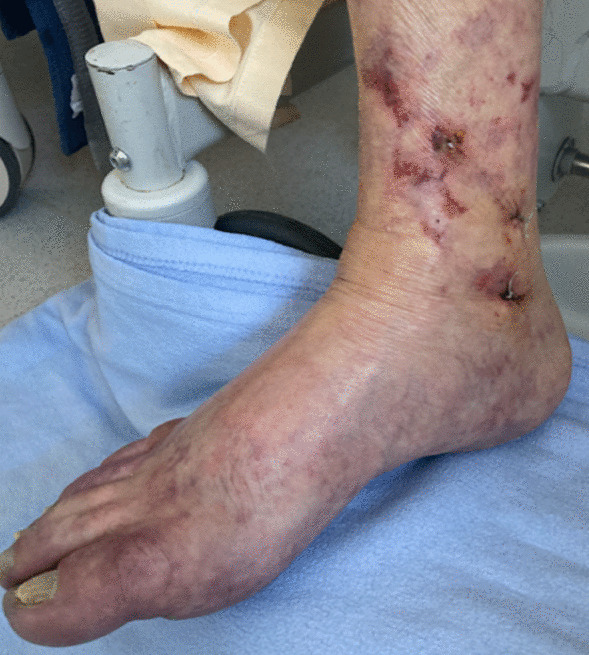
Vasculitis-like skin lesions on the right foot

Over the following week, skin lesions on both feet, consisting of livedo associated with distal necrosis, progressed (Fig. 2). Arterial doppler of lower limbs showed no macrovascular lesion. Skin biopsy found one focus of microvascular thrombosis but neither aspect of vasculitis nor inflammatory infiltrate. Search for serum cryoglobulinemia was positive with a dosage of 5.75 mg/L, which was made of oligoclonal immunoglobulins (Ig) G, A, and polyclonal M (type IIb). Cryocrit was inferior to 1%. Oligoclonal Ig dosage was at 25 g/L (type 2). Screening for hepatitis B and C was negative. Use of epinephrine and then norepinephrine infusion during the post-CPR phase was a possible contributor to the bilateral feet distal necrosis.

**Fig. 3:**
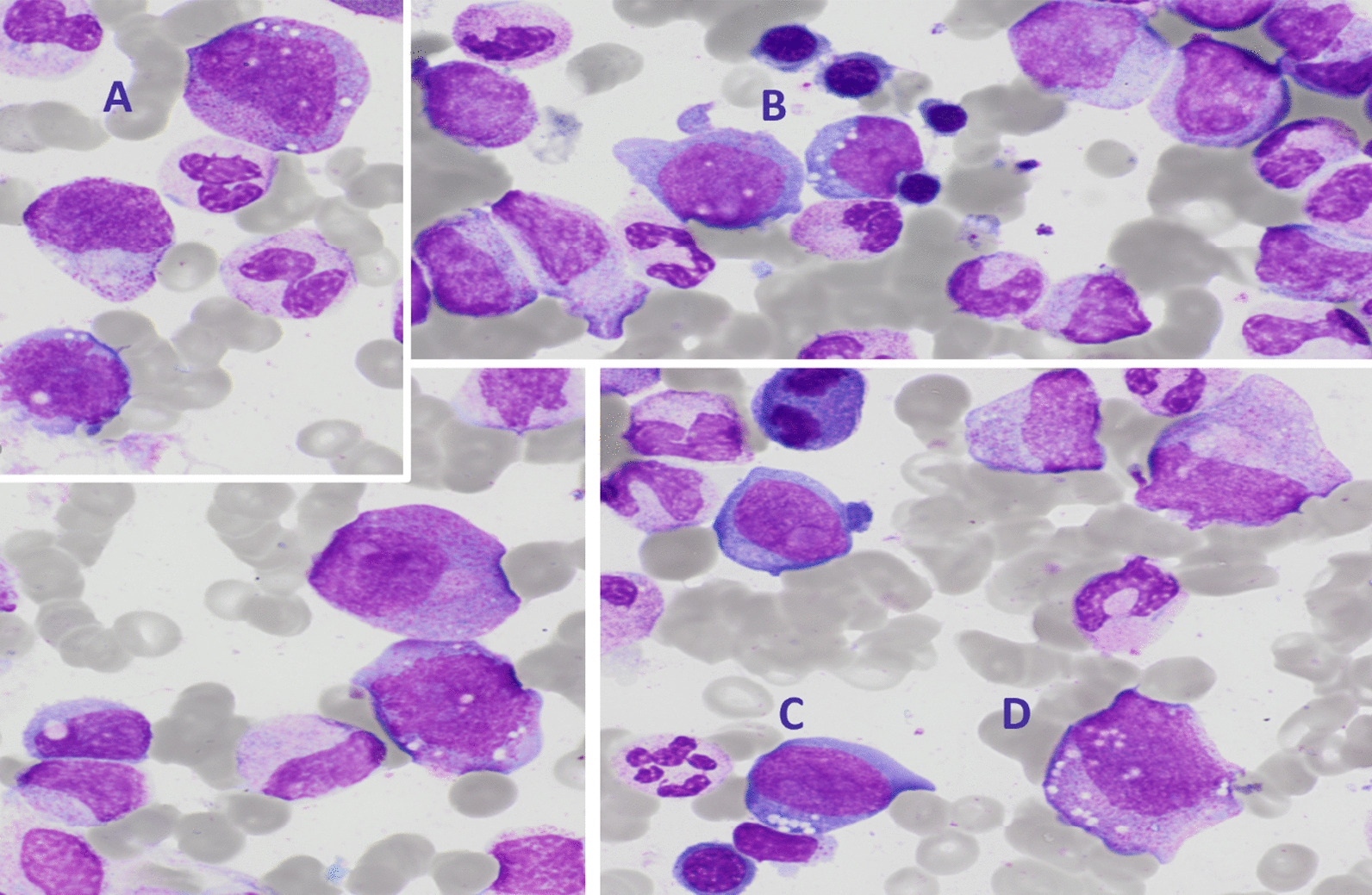
Bone marrow aspirate examination—bone marrow aspirate findings reveal characteristic vacuoles present in myeloid precursor cells: granular (**A**, **D**) and erythroïd (**B**, **C**) precursors—May–Grünwald–Giemsa stain ×10

**Fig. 4:**
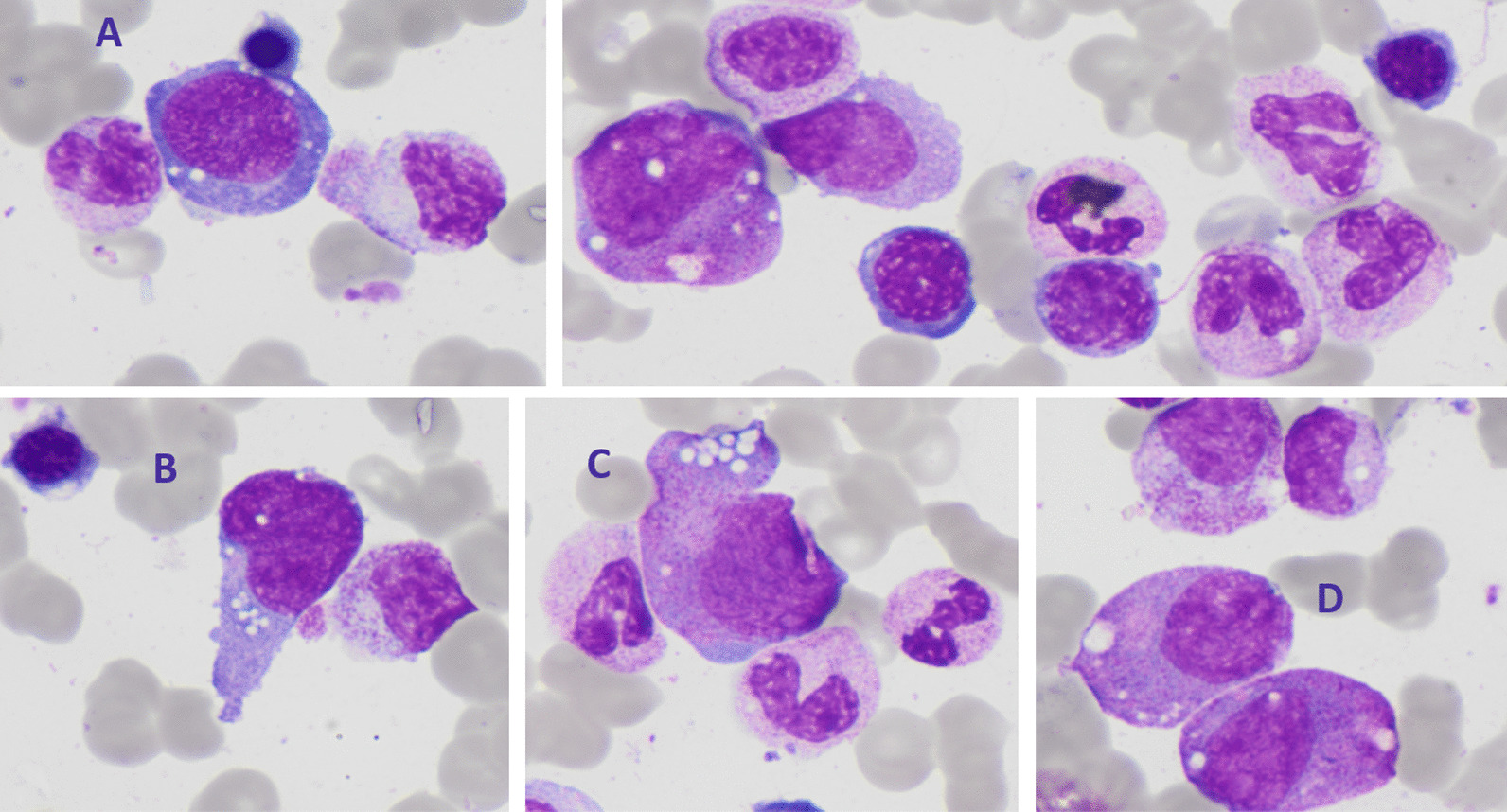
Bone marrow aspirate examination—bone marrow aspirate findings revealed characteristic vacuoles present in myeloid precursor cells: erythroid (**A**) and granular (**B**–**D**) precursors—May–Grünwald–Giemsa stain ×10

During the ICU stay, anemia, thrombocytopenia, and leukopenia developed over 2 weeks. Hemoglobin declined from 7.6 to 6.5 g/dL, platelets from 207 to 17 × 10^9^/L, and leukocytes from 4.2 to 1.6 × 10^9^/L. Ferritin was 3836 μg/L. A bone marrow aspirate showed signs of moderate dysplastic features, vacuoles in myeloid and erythroid precursors (Figs. 3, 4), no blast excess, and no argument for hemophagocytic lymphohistiocytosis. Myeloid next generation sequencing (NGS) panel did not find any classic myelodysplastic syndrome mutation.

At the same time, new deep vein thrombosis on the left leg appeared under heparin therapy, along with the onset of thrombocytopenia. A diagnosis of heparin-induced thrombocytopenia was confirmed with positive platelets aggregation test and positive PF4-heparin enzyme-linked immunosorbent assay (ELISA) test (4T score of 5). Switch to danaparoid sodium allowed for no recurrence of vein thrombosis.

Costal fractures following CPR delayed ventilation weaning. Finally, extubation on 29 January 2021 was successful.

Neurological complications such as delirium and swallowing disorders persisted. A new brain MRI was performed and was normal. A lumbar punction found no abnormality: absence of cells, proteinorachia of 0.45 g/L, normoglycorachia, no microorganism, negative *Cryptococcus* antigen test, and negative antineurones antibodies testing. Those residual neurological symptoms were interpreted as cardiac arrest sequelae.

**Fig. 5:**
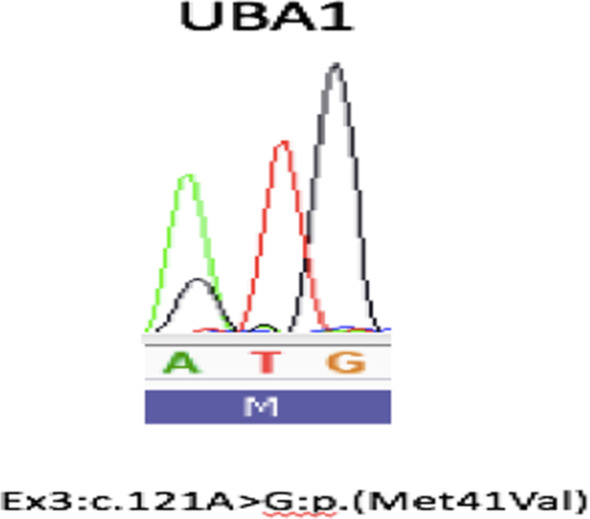
Genotype sequencing of the patient: *UBA1* mutation (p.Met41Val)

Following this complex symptomatology and based on the patient’s medical history (i.e., Sweet’s syndrome) investigation for a unifying explanatory autoinflammatory disease was initiated. Finally, testing for a genetic *UBA1* mutation in the patient’s blood came back positive (amino acid substitutions p.Met41Val), thus establishing the diagnosis of VEXAS syndrome (Fig. 5). High-dose corticosteroids (2 mg/kg/day of methylprednisolone) was started. It resulted in the stabilization of all symptoms, and allowed for ICU discharge. Further treatment was discussed during a multidisciplinary meeting and interleukin (IL) 1 receptor-1 inhibitor (Anakinra) was proposed to reduce the corticosteroid dose.

The patient was discharged and transferred to the internal medicine unit on 9 February 2021 after 1 month in the ICU. There, dysphagia improved rapidly.

Regarding the patient’s immunosuppressive treatment, oral corticosteroids were started on 1 February at 1 mg/kg and progressively decreased to 17.5 mg per day in March. Subcutaneous Anakinra 100 mg was started on 19 February. It was given initially once a day for 1 month and a half and progressively decreased over three weeks to once a week due to neutropenia recurrence. Clinically, skin lesions related to the Sweet’s syndrome reappeared during corticosteroid tapering.

During this hospitalization he presented with multiple infections: *Enterococcus faecalis* bacteriemia associated with sigmoid diverticulitis, hospital acquired pneumonia due to *Pneumocystis jirovecii*, and *Pseudomonas aeruginosa* and *Clostridium difficile* colitis. The patient finally fully recovered.

Right foot distal necrosis required a tarsometatarsal amputation of the right foot in early May, with left common iliac artery and right femoropopliteal artery angioplasty. Postoperative period was uneventful. The patient was transferred back from the surgical ward to the internal medicine unit a few days later. Physical rehabilitation was started and a slow decrease of corticosteroids dose continued, which was associated with resumption of Anakinra.

## Discussion and conclusion

To our knowledge, this is the first report of a diagnosis of VEXAS syndrome made in the ICU.

VEXAS syndrome stands for vacuoles, E1 enzyme, X-linked, autoinflammatory, and somatic syndrome. The syndrome behind this description is an autoinflammatory disease associated with myeloid dysplasia *UBA1* somatic mutation. Final diagnosis is made through genetic investigations.

Indeed, the history of this new syndrome has been highlighted in a recent *New England Journal of Medicine* article [8], which reported 25 cases of an adult-onset autoinflammatory disease with multiple organ manifestations occurring exclusively on men. Beck *et al*. reported that this new disease was linked to somatic mutations affecting methionine-41 (p.Met41) in *UBA1*, a major E1 enzyme that initiates ubiquitylation. Ubiquitylation is a type of posttranslational modification of proteins, used to regulate diverse aspects of cellular biology such as intracellular signaling and protein degradation through the proteasome or the lysosome system. It is performed through the actions of ubiquitin-activating enzymes (E1), ubiquitin-conjugating enzymes (E2), and substrate-specific ligases (E3), all three types of ubiquitin proteins included in a total of more than 600 enzymes. Regulation of ubiquitin signaling often occurs at the level of E2 and E3 enzymes. However, little is known regarding control of ubiquitin activation [2].

Somatic mutations in the *UBA1* gene (p.Met41) in the myeloid precursor cells result in the reduction of cytoplasmic *UBA1* functions in peripheral blood, leading to the activation of innate immune pathways (elevated interferon-γ, tumor necrosis factor (TNF), IL6, IL8) and to an upregulation of the cellular stress response. *UBA1* mosaic mutations have also been described in wild-type lymphocytes (T and B cells). Hence these mutations in ubiquitin activating enzymes (E1) could play an important role during hematopoiesis [9].

In the literature, patients with VEXAS syndrome present with systemic inflammation symptoms (polychondritis, Sweet’s syndrome, polyarteritis nodosa, giant cell arteritis), driven by mutant myeloid cells. This could imply that myeloid precursor cells could survive with these *UBA1* mutations and that mutant lymphocytes are negatively selected within bone marrow.

Two articles by Bourbon *et al*. [3] and van der Made *et al*. [10] describe the clinical characteristics of patients with VEXAS syndrome and the different therapeutic alternatives that were implemented. Our patient differs from the description in the literature in two aspects. First, they did not have an increased mean corpuscular volume compared with patients from Bourbon’s article, who presented with macrocytic anemia. Secondly, although corticosteroids remain the mainstay of the treatment in all patients, adjunct therapy used in our patient was an IL1 inhibitor, whereas in the literature drugs such as methotrexate, anti-TNFα, anti-IL6 receptor or calcineurin inhibitor were prescribed [3, 8]. All patients benefited from high-dose corticosteroids.

Our choice to start IL1 inhibitor was based on experts’ opinion, with underlying thoughts regarding patient presentation with important autoinflammatory features and without arguments for an acute hematology malignancy.

The heterogeneity of the VEXAS syndrome clinical presentations could be explained by the existence of different phenotypes, especially if those are associated with underlying hemopathy, thus leading to various therapeutic responses [1, 11]. This idea should be addressed and studied in the future reports.

Our patient’s story is complex, associated with many syndromes, and consequently investigations lasted for almost 2 years. A critical event (sudden cardiac arrest) without any discernable etiologies prompted us to collaboratively review the literature and search for a unifying diagnosis that would encompass all the recent patient’s symptoms and events. We hypothesized that the swallowing disorders led to acute obstruction of the upper airways, leading to a hypoxic cardiac arrest. This could have been worsened by the retro-cricoarytenoid edema highlighted during the end-of-year 2020 investigations.

We believe that the VEXAS syndrome was not directly responsible for the cardiac arrest per se, and the patient’s initial ICU management did not differ from other cardiac arrest cases. Nevertheless, the patient’s underlying autoinflammatory background is what made us wary and continue with immunology exams.

Our case clearly illustrates how the field of autoinflammatory diseases is yet to be fully understood. More importantly, the multidisciplinary interactions, including discussions and meetings, between all specialists (internal medicine, hematology, dermatology, molecular biology laboratory, and intensive care medicine) are the cornerstone of correct medical management of such complex patients with multisystem medical disorders. One point to highlight is that the patient was admitted in one of the few university hospitals in France where meetings on rare autoinflammatory disease occur, enabling complex debates to inform optimal patient care.

Post diagnosis and following high dose of corticosteroids, the patient stabilized, thus allowing for a rapid discharge after 1 month in a critical state in our unit. During his stay in the internal medicine ward, despite several severe infections, his general state improved, and autoinflammatory disorders were finally under control. Indeed, corticosteroids are part of the therapeutic management; however, the right adjunct immunosuppressant is still to be determined. In the future, further publications (case reports, case series, or cohort studies) may help to tailor the best treatment for each clinical situation.

It is likely that a certain proportion of male patients with relapsing polychondritis, Sweet’s syndrome, polyarteritis nodosa, or myelodysplastic syndrome could have underdiagnosed VEXAS syndrome [1, 12].

Hence, in the coming years, intensivists might have, under their care, similar male patients with autoimmune symptoms and no clear textbook diagnosis. The search for VEXAS syndrome may be an appropriate early differential diagnosis in these specific cases. Our case report raises the question of whether a quicker diagnosis, allowing for earlier start of targeted antiinflammatory treatment, could change and improve prognosis.

The patient signed a written informed consent.

## Data Availability

The datasets used and/or analyzed during the current study are available from the corresponding author on reasonable request.

## Abbreviations

CPR: Cardiopulmonary resuscitation
DVT: Deep vein thrombosis
ICU: Intensive care unit
MRI: Magnetic resonance imaging
VEXAS: Vacuoles, E1 enzyme, X-linked, autoinflammatory, and somatic
